# Treatment, outcome and re-vaccination of patients with SARS-CoV-2 vaccine-associated immune thrombocytopenia

**DOI:** 10.1007/s15010-022-01909-5

**Published:** 2022-10-04

**Authors:** Michael Ruzicka, Sonja Wurm, Lars Lindner, Martin Dreyling, Michael von Bergwelt-Baildon, Stefan Boeck, Clemens Giessen-Jung, Valeria Milani, Joachim H. Stemmler, Marion Subklewe, Oliver Weigert, Karsten Spiekermann

**Affiliations:** 1 Department of Medicine III, LMU Klinikum, LMU Munich, Marchioninistrasse 15, 81377 Munich, Germany; 2 Division of Hematology, Medical University of Graz, Graz, Austria; 3 Facharztzentrum Fürstenfeldbruck, Fürstenfeldbruck, Germany

**Keywords:** Immune thrombocytopenia, SARS-CoV-2, Vaccine-associated ITP, Post-vaccinal ITP, COVID-19 vaccine, COVID-19

## Abstract

**Purpose:**

Following the emergency use authorization of BNT162b2 by the Food and Drug administration (FDA) in early December 2020, mRNA- and vector-based vaccines became an important means of reducing the spread and mortality of the COVID-19 pandemic. The European Medicines Agency labelled immune thrombocytopenia (ITP) as a rare adverse reaction of unknown frequency after vector-, but not mRNA-vaccination. Here, we report on the long-term outcome of 6 patients who were diagnosed with de-novo, vaccine-associated ITP (VA-ITP), and on the outcome of subsequent SARS-CoV-2 re-vaccinations.

**Methods:**

Patients were included after presenting to our emergency department. Therapy was applied according to ITP guidelines. Follow-up data were obtained from outpatient departments. Both mRNA- or vector-based vaccines were each used in 3 cases, respectively.

**Results:**

In all patients, the onset of symptoms occurred after the 1st dose of vaccine was applied. 5 patients required treatment, 3 of them 2nd line therapy. All patients showed a complete response eventually. After up to 359 days of follow-up, 2 patients were still under 2nd line therapy with thrombopoietin receptor agonists. 5 patients have been re-vaccinated with up to 3 consecutive doses of SARS-CoV-2 vaccines, 4 of them showing stable platelet counts hereafter.

**Conclusion:**

Thrombocytopenia after COVID-19 vaccination should trigger a diagnostic workup to exclude vaccine-induced immune thrombotic thrombocytopenia (VITT) and, if confirmed, VA-ITP should be treated according to current ITP guidelines. Re-vaccination of patients seems feasible under close monitoring of blood counts and using a vaccine that differs from the one triggering the initial episode of VA-ITP.

**Supplementary Information:**

The online version contains supplementary material available at 10.1007/s15010-022-01909-5.

## Introduction

Coronavirus disease 2019 (COVID-19), a condition caused by the severe acute respiratory syndrome coronavirus 2 (SARS-CoV-2), has demonstrated a swift global spread after its outbreak in Wuhan, China in late 2019, and was finally declared a pandemic by the WHO in March, 2020 [1]. Following the emergency use authorization (EUA) of BNT162b2 (Comirnaty) by the Food and Drug administration (FDA) in early December 2020, several mRNA- as well as vector-based vaccines became a crucial, effective and safe cornerstone to tackle the global spread and reduce the mortality of COVID-19. Nevertheless, cases of vaccine-induced immune thrombotic thrombocytopenia (VITT), a rare and potentially fatal side effect, were described after immunization with the vector-based vaccine ChAdOx1-S/nCoV-19 (Vaxzevria), and eventually led to the discontinuation of its administration in March 2021 in some countries [2, 3]. New-onset or the exacerbation of immune-mediated disorders including immune thrombocytopenia (ITP) are known to occasionally occur secondary to some infectious diseases including SARS-CoV-2 infection or, less commonly, have been observed as rare side effects of certain vaccines [4–7]. Such vaccine-induced autoimmunity is believed to be caused by either the cross-reactivity between antigens or the immunostimulating effect of included adjuvants [8, 9]. Regarding SARS-CoV-2 vaccines, potential autoimmune side effects are known to be rare and the understanding of the underlying pathomechanisms, with the exception of VITT, remains limited [8–11]. There are few reports of de novo thrombocytopenia occurring after immunization with mRNA- as well as vector-based SARS-CoV-2 vaccines [12–15]. Due to the clinical presentation and response to corticosteroids and/or intravenous immunoglobulins (IVIGs), these postvaccinal phenomena show similarities to ITP [16]. The European Medicines Agency (EMA) reported on rare cases of ITP following vector-based vaccinations and recommended to update the product information of ChAdOx1-S/nCoV-19 and Ad26.COV2-S (Jcovden) to include ITP as an adverse reaction of unknown frequency [17, 18]. In patients with pre-existing ITP, it has been described that COVID-19 vaccinations with mRNA- and vector-based vaccines may result in the exacerbation of ITP in 12–25% of affected patients [15, 19, 20]. However, there are no reports up to date on patients with de novo ITP associated with SARS-CoV-2 vaccination receiving the re-vaccinations necessary to establish full or maintain immunity against COVID-19.

In Germany, SARS-CoV-2 vaccinations per day reached a peak between the end of March and mid-July, 2021 according to the Robert-Koch-Institut [21], exceeding 10^6^ vaccinations per day at given times. Here, we report about newly diagnosed cases of ITP in 6 patients after immunization with mRNA- or vector-based vaccines against SARS-CoV-2 within this time frame as well as consecutive re- or "booster" vaccinations, the latter referring to a third dose of vaccine or more.

### Patients and methods

**Fig. 1:**
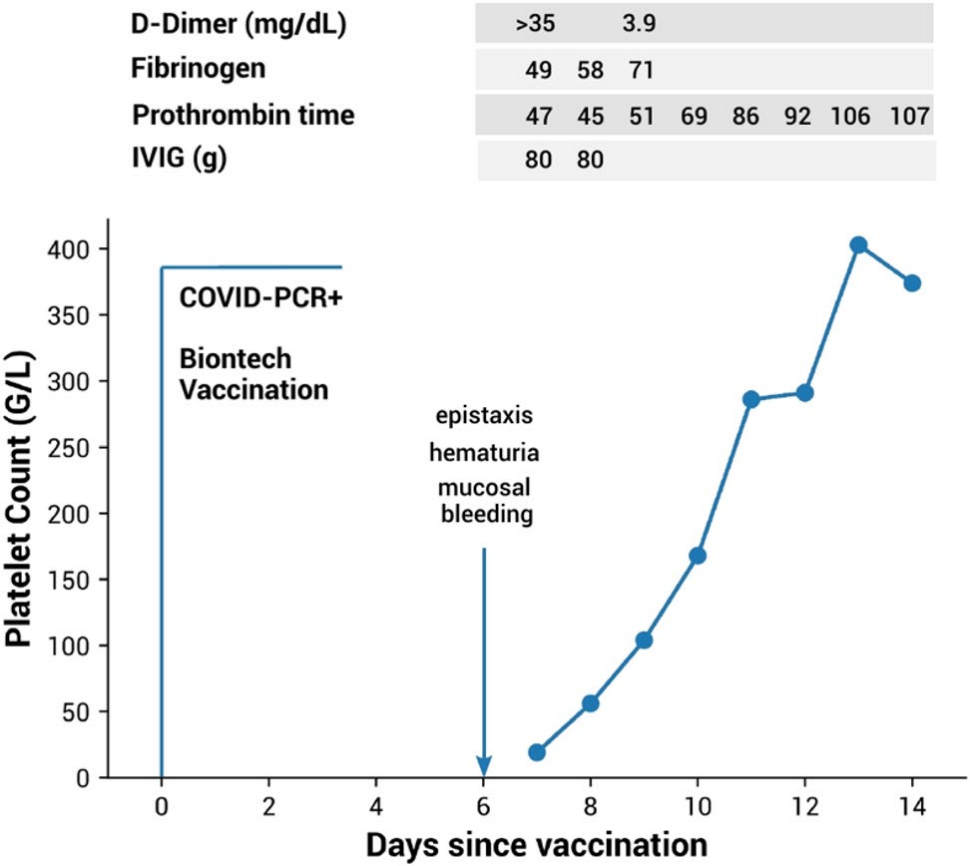
Response to therapy in patient 1. Day 0 is the time point of the first SARS-CoV-2 vaccination, simultaneously with which a SARS-CoV-2 infection was diagnosed by PCR test for the first time. The platelet count, laboratory parameters and treatment with IVIGs are depicted starting at the day of admission (day 7). Steroids (prednisolone 100 mg/day) were applied from day 6 to 19

**Fig. 2:**
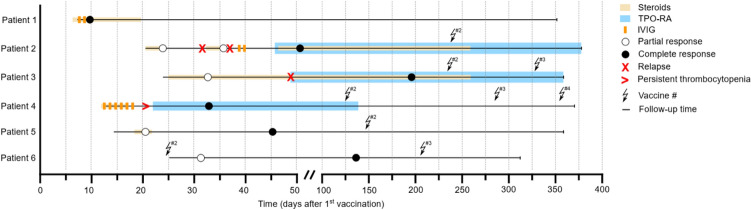
Treatment and course of disease including SARS-CoV-2 re-vaccinations after VA-ITP. Treatment of VA-ITP is depicted by colored bars. Partial and complete responses are indicated by circles. Relapse and persistent thrombocytopenia are reflected by the respective red symbols. Follow-up time, starting at admission, is visualized by the horizontal lines. SARS-CoV-2 re-vaccinations and their respective count are indicated by the serrated arrows

Patients were included after presenting to the emergency department of the LMU Klinikum, Munich, Germany, with suspected or confirmed vaccine-associated immune thrombocytopenia (VA-ITP). Data of the inpatient stay were used to characterize the initial course of the disease. Follow-up data were obtained from the LMU Klinikum outpatient department, oncologists in private practice or general practitioners. As there are no clinical guidelines on how to follow-up patients after VA-ITP up to date, the frequency of follow-up blood counts and clinical check-ups, including those after SARS-CoV-2 re-vaccination, was left to be decided by the treating physicians. ITP treatment responses were assessed as follows [22]: Complete remission (CR) is defined as a platelet count ≥ 100 × 10^9^/l and absence of bleeding; Partial response (PR) refers to a platelet count which doubles from the initial value and is between 30 and 100 × 10^9^/l in the absence of bleeding; Relapse is defined as a drop of the platelet count below 30 × 10^9^/l following a PR or CR. VITT was ruled out using a heparin-induced thrombocytopenia (HIT)-ELISA (Zymutest®) and, if not available, by clinical in conjunction with laboratory and radiological findings (D-dimer, computerized tomography angiography, sonography, etc.). Figures were created using R version 4.2.1 (Fig. 1), Inkskcape version 0.92 (Fig. 2) and GraphPad Prism version 9.3.0 (Supplemental Fig. 1).

## Results

### Clinical characteristics at admission

Between March and June 2021, 6 patients (3 men and 3 women) aged 41 to 83 years (median age: 68 years) presented with thrombocytopenia after vaccination against SARS-CoV-2 to our emergency department (Table 1). 5 out of 6 patients presented after receiving their first dose of vaccine, patient 6 presented after her second. 3 patients received the vector-based vaccine ChAdOx1-S/nCoV-19, the other 3 received the mRNA–based vaccine BNT162b2. The median time to onset of symptoms was 12.5 days, the median time to admission 17.5 days.

**Table 1:** Clinical data

Patient	1	2	3	4	5	6
Age (years)	70	41	76	83	60	66
Gender	m	f	f	m	m	f
1st vaccine	BNT162b2	ChAdOx1-S/nCoV-19	ChAdOx1-S/nCoV-19	BNT162b2	ChAdOx1-S/nCoV-19	Vaccine unknown
2nd vaccine, (day)	NA	BNT162b2 (237)	BNT162b2 (234)	Ad26.COV2-S (126)	BNT162b2 (147)	BNT162b2 (24)
3rd vaccine, (day)	NA	NA	BNT162b2 (329)	mRNA-1273 (282)	NA	mRNA-1273 (209)
4th vaccine, (day)	NA	NA	NA	mRNA-1273 (358)	NA	NA
Onset of symptoms^a^, day	5	10	21	5	15	22
WHO bleeding severity, grade	II	I	II	III	0	0
Admission, day	7	21	24	13	14	25
Start of therapy, day	6	21	25	12	18	NA

Day 0 is defined as the day of the 1st vaccination. *f* female; *m* male; *NA* not applicable; *ND* not determined. ^a^Symptoms included signs of bleeding and/or general symptoms (fatigue, headache, arthralgia, etc.)

4 patients presented with clinical signs of bleeding of varying severity to the emergency department. The other 2 patients presented due to generalized fatigue, arthralgia and headaches. Bleeding severity ranged from grade I to III according to the modified WHO Bleeding Scale [23] [Table 2] (Table 1). 3 out of 6 patients showed only minor signs of bleeding such as petechiae, mucosal hemorrhage and small hematomas. Patient 4 presented with clinically significant bleeding of grade III and required platelet transfusions. None of the 6 patients had a known medical history of immune thrombocytopenia, nor reported about bleeding events in the past. No lymphadenopathy, splenomegaly or other clinical signs suggestive of an underlying malignant disease were found in the physical examination. None of the patients had a known history of rheumatologic or immunologic disorders.

**Table 2:** Laboratory parameters at admission

Patient	1	2	3	4	5	6
Platelet count at baseline, × 10^9^/l (days before 1st vaccination)	170 (–201)	244 (–232)	226 (–158)	185 (–79)	220 (–458)	233 (–1132)
Platelet count at admission, × 10^9^/l	19	3	17	1	28	27
Anti-platelet antibodies	neg	GP Ia/IIa, GP Ib/IX	GP IIb/IIIa	GP IIb/IIIa, GP Ib/IX	neg	GP IIb/IIIa, GP Ib/IX
Lupus antikoagulant	ND	neg	ND	neg	neg	neg
Heparin/PF4 antibody rapid test	pos	pos	neg	neg	neg	neg
HIT-IL-acustar-assay	neg	neg	neg	neg	neg	neg
HIT-elisa (Zymutest®)	neg	neg	neg	ND	neg	neg

*GP* glycoprotein*; HIT* heparin-induced thrombocytopenia; *NA* not applicable; *ND* not determined; *neg.* negative; *pos* positive

### Laboratory and further findings at admission

Laboratory test results at admission are shown in Table 2. All patients underwent SARS-CoV-2 PCR testing. 5 out of 6 patients had negative results. A known SARS-CoV-2 infection was confirmed in patient 1 by PCR testing, who was diagnosed with COVID-19 on the day of his first vaccination 7 days ahead of admission. Notably, he was asymptomatic at the time of vaccination, 10 days prior to which he experienced a mild cough. At the time of admission all 6 patients showed thrombocytopenia with a median platelet count of 18 × 10^9^/l (Table 2). Pseudothrombocytopenia was excluded in all patients. In 2 out of 6 cases we observed isolated thrombocytopenia with regular values of plasmatic coagulation. Levels of D-dimer were slightly elevated in three patients, but without further signs of disseminated intravascular coagulation (DIC). Patient 1 presented with bicytopenia (leucocytes: 2,2 × 10^9^/l, platelets: 19 × 10^9^/l) and severe coagulopathy after receiving his first dose of BNT162b2 while also testing positive for SARS-CoV-2 on the day of vaccination (Fig. 1). The coagulopathy was classified as DIC with low levels of fibrinogen (49 mg/dl), elevated D-dimer (> 35 mg/dL) and decreased prothrombin time (47%). Further, low levels of haptoglobin < 0,10 g/l in combination with elevated levels of LDH (2108 U/l) indicated ongoing hemolysis. The Coombs test was negative. VITT was excluded in all 6 patients as described in patients & methods. Computerized tomography angiography (CTA) was performed in 3 patients without any signs of sinus venous thrombosis. Pre-vaccination platelet counts dating back 79 to 1123 days were within the normal range (Table 2). The clinical and laboratory findings, including positive anti-platelet antibodies in 4 out of 6 patients, are indicative of ITP (Table 2). A diagnostic work-up for underlying malignant, infectious and immune-mediated conditions was performed in all patients and found no other potential triggers of ITP.

### Treatment and response

Patient 1 was diagnosed with pneumonia while hospitalized and treated antibiotically as common in bacterial superinfections secondary to COVID-19. In 5 out of 6 patients, treatment with steroids was commenced (Fig. 2). 2 patients received dexamethasone for 4 days (patient 5: 20 mg/day, patient 2: 40 mg/day). Patients 1, 3 and 4 were given prednisolone (1 mg/kg bw/day, max. 100 mg/day). Patient 4 was continued to be treated with dexamethasone (40 mg/day) instead after 2 days of receiving prednisolone. Patient 6 did not receive any therapy.

The follow-up time ranges from 288 to 359 days (Table 3). For follow-up, day 0 was defined as the day of treatment initiation, in patient 6 (receiving no treatment) as the day of admission. Regarding the platelet response (Fig. 2), a very heterogeneous pattern was observed. Patients 1 and 5 showed a CR on days 3 and 27, respectively, after both received steroids and patient 1 additionally received intravenous immunoglobulins (IVIGs; 80 g in total; Figs. 1 and 2). Patient 4 showed persistent thrombocytopenia (< 10 × 10^9^/l) after 1 week of steroid treatment, with ongoing signs of bleeding requiring platelet transfusions and IVIGs (Fig. 2). A 2nd line therapy with a thrombopoietin receptor agonist (TPO-RA; Romiplostim 1 mcg/kg bw/week) was started on day 10, with a consecutive CR on day 21. Patients 2 and 3 initially showed partial responses (PR) under steroids, followed by relapses requiring treatment with TPO-RA. Patient 2 relapsed twice after dexamethasone therapy (40 mg/day for 4 days), and finally achieved a CR shortly after treatment with Eltrombopag (50 mg/day) was commenced. In patient 3, Eltrombopag was initiated after the first relapse at a dose of 25 mg/day and increased up to 50 mg/day, also leading to a CR. After a spontaneous partial remission, patient 6 underwent off-label treatment with high-dose vitamin C and acetylsalicylic acid at her own request. At the end of follow-up time, all patients were in CR with stable platelet counts, with patients 2 and 3 still depending on TPO-RA.

**Table 3:** Therapy and response

Patient	1	2	3	4	5	6
Last response	CR	CR	CR	CR	CR	CR
Lines of therapy	1	2	2	2	1	0
Follow-up time, days^a^	346	357	336	359	343	288
Ongoing therapy at last day of follow-up	NA	TPO-RA	TPO-RA	NA	NA	NA
Platelet count, × 10^9^/l at last day of follow-up	429	172	245	204	221	230

*CR* indicates complete response; *TPO-RA* thrombopoietin receptor agonist. ^a^Day 0 was defined as the first day of therapy, in patient 6 as the day of admission

### Re-vaccinations against SARS-CoV-2

In the further course 5 of the 6 patients received up to 3 more doses of SARS-CoV-2 vaccines. To decrease the risk of another episode of VA-ITP, none of the patients were re-vaccinated with the vaccine triggering the initial episode of VA-ITP. Instead, vaccines of different manufacturers were used (BNT162b2, Ad26.COV2-S, mRNA-1273; Table 1). With the exception of patient 2, no significant changes in platelet counts were observed in the weeks and months hereafter (Fig. 2). Of note, patients 2 and 3 were under ongoing treatment with TPO-RA at this time. In patient 2, who received the second dose of BNT162b2 on day 237 and demonstrated stable platelet counts in the following days, a dose reduction step of TPO-RA was undertaken on day 255 at the patients request (Supplemental Fig. 1). On day 264, the blood count revealed a platelet drop to 25 × 10^9^/l, which recovered swiftly to normal values after the TPO-RA dose was re-escalated.

## Discussion

VA-ITP in general is a known but rare phenomenon [24–27]. While there are reports about ITP occurring after immunization with SARS-CoV-2 vaccines, knowledge of causality, actual incidence and importantly guidelines for management of treatment are still limited. Here, we reported about newly diagnosed ITP in 6 patients after the immunization with mRNA- or vector-based vaccines against SARS-CoV-2 and consecutive “booster” or re-vaccinations.

In a large case-series study, data from the Vaccine Adverse Event Reporting System (VAERS) of the United States (US) revealed 15 cases of thrombocytopenia after immunization with BNT162b2 and 13 cases of thrombocytopenia after mRNA-1273 in a total of more than 35 million doses of applied SARS-CoV-2 vaccines [12]. Interestingly, the number of postvaccinal thrombocytopenia cases did not exceed the expected incidence rate of ITP within the general population of the US. Therefore, one main question remains if there is an actual causal relation between SARS-CoV-2 vaccination and the onset of ITP, or if the adverse events we observed in our patients resemble episodes of primary ITP diagnosed coincidentally after vaccination. Although pre-vaccination platelet counts were within the normal range in all 6 of our patients, the test results of patients 5 and 6 date back 1.5 and even 3 years, respectively (Table 2). Even though pre-existing ITP cannot be ruled out with full certainty, the close temporal relation of vaccination and new-onset of bleeding signs in 4 of the 6 patients remains indicative of a causal link. The timing of symptom onset in general is another point to consider, as all patients noticed symptoms after the first dose of the respective vaccine was applied. These observations are in line with investigations by Lee et al. [15], who analyzed data sets of 77 patients with new-onset ITP after mRNA-vaccination included in the VAERS. The authors report of a median time to disease onset of 8 days. The majority of patients (77.3%) presented after the first dose. In an observational study of 34 patients with chronic or persistent thrombocytopenia by Jiang et al. [28], 14 patients showed a post-vaccination platelet decrease, the majority of which occurred after the 1st immunization as well. Interestingly, after the 2nd immunization, levels of platelets remained stable. For a purely coincidental, temporal relation between new-onset ITP and SARS-CoV-2 vaccination, a more equal distribution of disease onset between the first and second dose of vaccine would be expected.

Recently, the EMA recommended to update the product information of the vector-based vaccines ChAdOx1-S/nCoV-19 and Ad26.COV2-S due to rare cases of immune thrombocytopenia occurring within the first weeks after their administration [17]. In the 3 ITP patients of our cohort who received ChAdOx1-S/nCoV-19, the onset was within 4 weeks as described in the statement by the EMA [17]. In contrast, the literature addressing thrombocytopenia following mRNA vaccines is scarce, as only few cases have been reported up to this date [12]. An interesting observation was made in a large retrospective data analysis by Lee et al. [29]. Apart from a total of 77 suspected cases of de-novo ITP identified in the VAERS, exacerbation of pre-existing ITP occurred in 19 of 109 cases after SARS-CoV-2 immunization with BNT162b2 or mRNA-1273 vaccines. Taking these observations into account, both de-novo ITP or the exacerbation of pre-existing subclinical ITP may explain the thrombocytopenia in our patients following the vaccination with BNT162b2 in particular.

To this date there are no clear recommendations regarding the treatment of VA-ITP. Evaluating the treatment response in our cases, all 5 patients who received ITP-guided therapy reached a CR. Patient 6 did not receive any therapy and showed a spontaneous PR after 24 days and a CR in the later course. Patient 1, who tested positive for SARS-CoV-2 on the day of his first immunization with BNT162b2, responded well to steroids and only 2 doses of IVIGs, showing a normalization of platelet counts as well as coagulation parameters within less than a week (Fig. 1). The time interval between his immunization and the onset of bleeding signs and thrombocytopenia was 7 days, which is in line with the time frame of reported ITP cases following vaccination with BNT162b2 [29]. The fast response to IVIGs suggests an immunologic pathomechanism of platelet consumption as it occurs during DIC, although no anti-platelet antibodies were detected in this particular case. Due to the temporal overlap of SARS-CoV-2 infection and vaccination, we cannot fully rule out a COVID-19 associated DIC/thrombocytopenia in this case.

Even though all our patients achieved a CR, 3 out of 5 required 2nd line therapy with TPO-RA (Fig. 2). Similarly, Paulsen et al. [30] describe a case series of 4 patients with newly diagnosed ITP after ChAdOx1-S/nCov-19 vaccination, in which ITP-guided 1st line therapy with steroids and IVIGs also led to CR in all cases. One patient with a history of auto-immune disorders experienced a relapse, which was overcome by 2nd line therapy with TPO-RA, leading to a stable CR eventually [30]. Likewise, Lee et al. [29] report on an almost 90% response rate to 1st line treatment in a retrospective cohort of 28 cases of postvaccinal de novo ITP. Patients of this cohort in need of 2nd line treatment showed improvement after TPO-RA, vincristine or rituximab treatment. In 16 cases of exacerbated, pre-existing ITP receiving rescue therapy, all patients responded with platelets rising > 30 × 10^9^/l or return to pre-vaccination ranges. Rescue therapies included corticosteroids, IVIGs, TPO-RA or a combination of IVIGs, corticosteroids, rituximab and cyclosporine. Taken together, most patients affected by VA-ITP seem to respond well to 1st line therapy, while a few require 2nd line options. The overall prognosis and (long term) relapse rates cannot be accurately estimated at this point and require future research.

5 of our patients received up to 3 consecutive doses of SARS-CoV-2 vaccines after the initial episode of VA-ITP (Fig. 2). None of the patients were re-vaccinated using the vaccine initially triggering the VA-ITP. Instead, vaccines of different manufacturers were applied (Table 1). Interestingly, we observed no significant changes in platelet counts in the weeks hereafter with the exception of patient 2 (Supplemental Fig. 1). In the latter, due to a temporal overlap of both re-vaccination and dose reduction of TPO-RA, the actual cause for the platelet drop remains unclear or may even be promoted by both events. However, based on the kinetics of the platelet drop and the consecutive recovery after first dose reduction and then re-escalation of TPO-RA, we do think that the vaccination is less likely to account for these events. There is no published literature on COVID-19 “booster” or re-vaccinations in patients with de novo ITP after SARS-CoV-2 vaccination to the authors’ knowledge so far. In a meta-analysis by E.-J. Lee et al. [15], 19 out of 117 patients with de novo or preexisting ITP, but not SARS-CoV-2 VA-ITP, experienced an exacerbation after the first and 14 out of 70 after the second dose of either BNT162b2 or mRNA-1273. Over half of the patients with a platelet drop after the first dose had stable or increased platelet counts after the second. In a prospective cohort study by Visser et al. [19], 30 of 218 patients (13.8%) with pre-existing ITP experienced an exacerbation after 1 or 2 doses of mostly mRNA-1273. Despite one fatality, most patients responded well to ITP-guided therapy. Even though our patient number is small, taken together with these results it appears as if “booster” or re-vaccinations may be considered in patients with a previous episode of SARS-CoV-2 VA-ITP if blood counts are carefully monitored consecutively. However, it seems reasonable to use a different SARS-CoV-2 vaccine than the one triggering the initial episode of VA-ITP.

In summary, we observed 6 cases of VA-ITP occurring within 4 weeks after immunization against SARS-CoV-2 with both mRNA- and vector-based vaccines, respectively. 5 patients were treated with ITP-guided 1st line therapy, 3 of them later needed 2nd line treatment. Although some patients showed severe courses of disease, a CR could eventually be achieved in all patients. Of note, 2 patients still require TPO-RA at the end of follow-up time to maintain a stable CR. 5 of the patients received up to 3 consecutive doses of SARS-CoV-2 vaccines after the initial episode of VA-ITP without severe complications. VA-ITP remains a very rare phenomenon, with calculations based on the VAERS database showing that the incidence rate of VA-ITP after mRNA-based SARS-CoV-2 vaccinations does not exceed the background incidence of ITP in the general adult population [12]. Yet, awareness needs to be raised for ITP as a potential differential diagnosis in patients presenting with signs of bleeding and/or thrombocytopenia following vaccination against SARS-CoV-2. Patients with pre-existing ITP should be monitored closely within the first weeks after vaccination. It also seems as if patients with a history of VA-ITP after SARS-CoV-2 vaccination may be re-vaccinated using a SARS-CoV-2 vaccine differing from the one initially triggering the VA-ITP episode. This should only be undertaken under close monitoring of blood counts, and more studies are needed to prove this approach safe. Nevertheless, the benefits of both mRNA- as well as vector-based SARS-CoV-2 vaccines are well established and continue to be the most important cornerstone in the fight against the COVID-19 pandemic.

## Supplementary Information

Below is the link to the electronic supplementary material.

Supplementary file1 (PDF 204 KB)

## Abbreviations

Bw: Body weight
COVID-19: Coronavirus disease 2019
CR: Complete response
CTA: Computerized tomography angiography
DIC: Disseminated intravascular coagulation
EMA: European medicines agency
EUA: Emergency use authorization
FDA: Food and drug administration
ITP: Immune thrombocytopenia
IVIGs: Intravenous immunoglobulins
PR: Partial response
PF4: Platelet factor 4
SARS-CoV-2: Severe acute respiratory syndrome coronavirus 2
TPO-RA: Thrombopoietin receptor agonists
US: United states
VAERS: Vaccine adverse event reporting system
VA-ITP: Vaccine-associated ITP
VITT: Vaccine-induced immune thrombotic thrombocytopenia
