# Supplementary material for: Treatment, outcome and re-vaccination of patients with SARS-CoV-2 vaccine-associated immune thrombocytopenia
Source: Infection. 2022 Oct 4;51(1):231–8. doi: 10.1007/s15010-022-01909-5 (PMC9531644; doi:10.1007/s15010-022-01909-5)
Supplement: Supplementary file 1 — Supplementary file1 (PDF 204 KB) [file 15010_2022_1909_MOESM1_ESM.pdf]

Article Title:

Treatment, outcome and re-vaccination of patients with SARS-CoV-2 vaccine-associated immune thrombocytopenia

Journal: Infection

Authors:

Michael Ruzicka, Sonja Wurm, Lars Lindner, Martin Dreyling, Michael von Bergwelt-Baildon, Stefan Boeck, Clemens Giessen-Jung, Valeria Milani, Joachim H. Stemmler, Marion Subklewe, Oliver Weigert and Karsten Spiekermann

Corresponding author:

Karsten Spiekermann

[1]*Department of Medicine III, University Hospital, LMU Munich, Munich, Germany*

E-mail: Karsten.Spiekermann@med.uni-muenchen.de

Supplemental materials

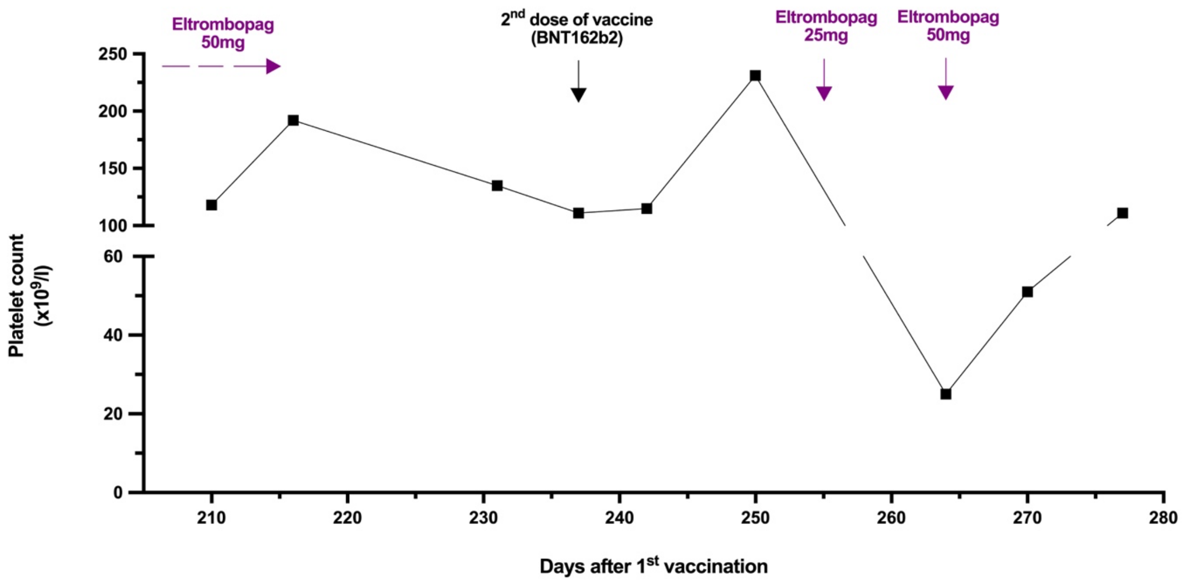

Supplemental Figure 1. Platelet counts of patient 2 after re-vaccination with BNT162b2. The patient was re-vaccinated while under treatment with 50mg Eltrombopag per day. The time points of re-vaccination and Eltrombopag dose adjustments as well as thrombocyte counts over time are displayed in the figure.
